# Fushiming Capsule Attenuates Diabetic Rat Retina Damage via Antioxidation and Anti-Inflammation

**DOI:** 10.1155/2019/5376439

**Published:** 2019-07-18

**Authors:** Mengshan He, Pan Long, Lunfeng Guo, Mingke Zhang, Siwang Wang, Hongling He

**Affiliations:** ^1^Department of Chinese Material Medical and Natural Medicines, Fourth Military Medical University, Xi'an, Shaanxi 710032, China; ^2^Center of Clinical Aerospace Medicine, Fourth Military Medical University, Xi'an, Shaanxi 710032, China; ^3^Department of Pharmacy, Central Hospital of Ankang City, Ankang 725000, Shaanxi, China; ^4^Xi'an Lejian Biological Technology Co., Ltd., Xi'an 710032, Shaanxi, China; ^5^Academic Journals Publishing Center of Education Department, The First Affiliated Hospital of Xi'an Jiaotong University, Xi'an 710061, Shaanxi, China

## Abstract

**Aims:**

Diabetic retinopathy (DR) remains one of the leading causes of acquired blindness. Fushiming capsule (FSM), a compound traditional Chinese medicine, is clinically used for DR treatment in China. The present study was to investigate the effect of FSM on retinal alterations, inflammatory response, and oxidative stress triggered by diabetes.

**Main Methods:**

Diabetic rat model was induced by 6-week high-fat and high-sugar diet combined with 35 mg/kg streptozotocin (STZ). 30 days after successful establishment of diabetic rat model, full field electroretinography (ffERG) and optical coherence tomography (OCT) were performed to detect retinal pathological alterations. Then, FSM was administered to diabetic rats at different dosages for 42-day treatment and diabetic rats treated with Calcium dobesilate (CaD) capsule served as the positive group. Retinal function and structure were observed, and retinal vascular endothelial growth factor-*α* (VEGF-*α*), glial fibrillary acidic (GFAP), and vascular cell adhesion protein-1 (VCAM-1) expressions were measured both on mRNA and protein levels, and a series of blood metabolic indicators were also assessed.

**Key Findings:**

In DR rats, FSM (1.0* *g/kg and 0.5* *g/kg) treatment significantly restored retinal function (a higher amplitude of b-wave in dark-adaptation 3.0 and OPs2 wave) and prevented the decrease of retinal thickness including inner nuclear layer (INL), outer nuclear layer (ONL), and entire retina. Additionally, FSM dramatically decreased VEGF-*α*, GFAP, and VCAM-1 expressions in retinal tissues. Moreover, FSM notably improved serum antioxidative enzymes glutathione peroxidase, superoxide dismutase, and catalase activities, whereas it reduced serum advanced glycation end products, methane dicarboxylic aldehyde, nitric oxide, and total cholesterol and triglycerides levels.

**Significance:**

FSM could ameliorate diabetic rat retina damage possibly via inhibiting inflammation and improving antioxidation.

## 1. Introduction

Diabetic retinopathy (DR) is a kind of neurovascular complication of diabetes [1]. Despite the availability of effective treatment, DR remains one of the most common causes of blindness among working age adults [2]. It is expected that the global number of diabetes patients will double by 2030 comparing with about 400 million in 2017 [3, 4]. Furthermore, more than a third of people with diabetes have symptoms of DR [5]. Currently, the main effective methods for DR treatment include laser photocoagulation, vitrectomy, and intravitreal injections of corticosteroids or anti-VEGF agents [2, 6]. These mentioned methods exhibit modest clinical effects, but none is yet to fully deaden clinical progression or reverse retinal damage [7]. Additionally, the frequent intraocular injections probably cause side effects involving corneal scarring and intraocular infection, not to mention the expenses related to frequent ophthalmology clinician visits. The alternative solution is limited for patients, which makes it an urgency to find new medications.

Fushiming capsule (FSM), a compound traditional Chinese medicine, is composed of bioactivity components, such as puerarin, ginsenosides Rb1, ligustilide, aurantio obtusin, and chrysophanol [8]. Puerarin, being a vasodilator [9, 10], can reduce the oxidative stress damage of the retina [11, 12] and protect the retina through inhibiting inflammation and neuronal damage induced by proinflammatory factors [13, 14]. Ginsenosides Rb1 exhibits neuroprotective potential [15] and cytoprotective effects against oxidative stress-mediated apoptosis and other neuronal disturbances [16]. It is reported that ligustilide, which is responsible for the major bioactivities of Angelica sinensis, presents anti-inflammatory [17] and antioxidative [18] potential and prevents accumulation of platelets in blood vessels [19]. Aurantio obtusin, the major bioactivity component of cassia seed [20, 21], has various of biological properties, such as antioxidation, anticoagulating, and antihypertension [22, 23]. According to previous researches, Chrysophanol had antioxidative, anti-inflammatory, antiaging, and neuroprotection effects in Alzheimer disease (AD), cardiac injury, and diabetes [24–28]. Additionally, lycium barbarum and whitmania pigra are also constituents of FSM [8]. Thereinto, lycium barbarum could attenuate hypoxia and mitochondrial stress [29] and protect the function and viability of the retinal cells [30] in DR. Besides, whitmania pigra is applied clinically as a traditional Chinese medicine because of its anticoagulant and antithrombosis effects. Hence, this compound traditional Chinese medicine has potentially specific benefits for regulating the vascular microenvironment and resisting oxidation and inflammation.

It is well known that Calcium dobesilate (calcium 2,5-dihydroxybenzenesulfonic acid, CaD) has been prescribed for decades to some diabetic patients in the early stage of DR to stave off its progression [31]. According to the previous study, this synthetic compound exhibited systemic anti-inflammatory and antioxidative properties in the treatment of microcirculatory disorders [32]. Therefore, in this work, CaD was administered in the positive group, for 42 days, to diabetic animals, after 30 days of diabetes.

Among the factors implicated in the pathogenesis of DR, hyperglycemia-mediated inflammation and oxidative stress are considered as major relevant instigators of DR [33]. Inflammatory process, such as cytokine network and leukocyte adhesion (e.g., vascular cell adhesion protein-1 (VCAM-1)), induces vascular hyperpermeability and blood-retinal barrier breakdown [34]. Oxidative stress can directly or indirectly stimulate the release of proinflammatory cytokines, vascular endothelial growth factor-*α* (VEGF-*α*), and nitric oxide (NO), which results in retinal cell damage, pathological neovascularization, and the succeeding development of DR pathogenesis. Additionally, inflammation responses and low activities of antioxidative enzymes such as glutathione peroxidase (GSH-Px), Catalase (CAT), and superoxide dismutase (SOD) contribute to further increasing accumulation of reactive oxygen species (ROS) [35, 36]. This results in enhanced oxidative stress and aggravated retinal neuron damage and cell apoptosis. Moreover, hyperglycemia also causes Müller cells to produce proinflammatory cytokines to restore the retinal homeostasis [37, 38]. This is characterized by upregulation of glial fibrillary acidic (GFAP) and VEGF, which leads to a glial reaction and blood barrier hyperpermeability [39].

Based on the above observations, the present study was designed to test whether Fushiming capsule, a specific compound traditional Chinese medicine, could alleviate diabetic retina damage induced by high-fat and high-sugar diet combined with a low dose STZ injection and to determine how Fushiming capsule modulates oxidative stress and inflammatory responses.

## 2. Materials and Methods

### 2.1. Animals

Male Sprague Dawley (SD) rats (6-8 weeks old, 180-220 g) were obtained from the Laboratory Animal Center of Fourth Military Medical University in Xi'an, Shaanxi Province, China (license No.2014270138S). Rats were maintained under standard laboratory conditions, with 23°C ± 3°C room temperature, 40-65% humidity, and 12 h light/dark cycles, and were provided with ad libitum. All experiments were performed in accordance with the ARVO Statements for the use of Animals in Ophthalmic and Vision Research. All protocols were approved by the research ethics committee for care of laboratory animals at Fourth Military Medical University and experiments were conducted in compliance with its guidelines for experimental animals.

### 2.2. Diabetic Retinopathy Rat Model and Drug Administration

SD rats were fed with high-fat and high-sugar diet (breeding rodent material 54.6%, lard 16.9%, sucrose 14%, casein 10.2%, premix 2.1%, and maltodextrin 2.2%) (Slac Laboratory Animal, Shanghai, China) for 6 weeks after the rats were allowed to acclimate for 3 days. Then, the diabetic rat model was induced by a single intraperitoneal injection of streptozotocin (35 mg/kg body weight [40]) (Sigma, USA) after 6-week high-fat and high-sugar diet. Seventy-two hours after STZ injection, blood glucose level was measured by ACCU-CHEK Performa (Roche, Germany) and rats showing a blood glucose level above 16.7 mmol/L were considered as diabetes mellitus and selected for the study feeding with high-fat and high-sugar diet. After 30 days, ERG and OCT were performed for observing pathological changes in diabetic retinas. Then, according to the amplitude of the electroretinogram OPs2 wave, 40 successfully constructed diabetic retinopathy (DR) model rats were selected and randomly divided into 5 groups (n=8): untreated diabetic model group (normal saline, equal volume), positive group (calcium dobesilate (CaD) capsule, 0.2 g/kg, Guizhou Tian'an Pharmaceutical Co., Ltd., China) (CaD group), FSM high-dose group (FSM, 1.0 g/kg, Xi'an Lejian Biological Technology Co., Ltd., China), FSM medium-dose group (FSM, 0.5 g/kg), and FSM low-dose group (FSM, 0.25 g/kg), and different treatments were given for 42 consecutive days. In addition, 8 normal rats with matched ages were selected as the normal group (normal saline, equal volume) and were fed with ordinary diet. The method of administration is gavage, once a day. The rats were monitored throughout the study for body weight and blood glucose and were maintained on their respective diets throughout the treatment period.

### 2.3. Full Field Electroretinography (ffERG)

Full field electroretinography (ffERG) measurements were obtained at 30 d after diabetic rat model established and 42 d posttreatment (72 d of total duration of diabetes). ERG operation procedures were performed like the method described previously [41, 42] which was in compliance with the ISCEV guidelines. The ffERG recording items covered dark-adapted 0.01 response, dark-adapted 3.0 response, dark-adapted oscillatory potential response, light-adapted 3.0 response, and light-adapted flicker response. Specifically, animals were placed in a dark environment for dark adaption overnight (>12 h) and prepared for recording under a dim red-light condition. Anesthesia was applied with intraperitoneal (IP) injection of 3 mL/kg 1% sodium pentobarbital (Sigma, USA) and 50 *μ*L sumianxin II (Jilin Shengda Animal Pharmaceutical Co., Ltd., China). The pupils were dilated with 0.5% tropicamide solution (Shenyang Xingji Corporation, China). FfERG was recorded using the full-field (Ganzfeld) stimulation and a computer system (RETI port, Roland Consult GmbH, Germany) with custom-made silver chloride electrodes. The active electrode was a ring electrode placed at the center of the cornea. Stainless steel needle electrodes were placed in the cheek and tail to serve as the reference and ground leads, respectively. Levofloxacin eye drops (Suzhou, China) were used three times a day after ERG testing to avoid infection.

### 2.4. Optical Coherence Tomography (OCT)

Optical coherence tomography (OCT) images were taken to observe structural changes of the retina at 30 d after diabetic rat model established and 42 d posttreatment (72 d of total duration of diabetes). OCT scans were performed with a Micron IV fundus camera and an OCT Scan Head equipped with a mouse objective lens. Linear OCT scans consisted of a series of 1024 single point A-Scans. Rat eyes had previously been dilated with 0.5% tropicamide. And hydroxyl ethyl cellulose l (Bausch & Lomb Freda, China) was used as a coupling gel. Fundus and OCT images were captured from 20 positions for each eye using a Retinal Imaging System (OPTO-RIS, OptoProbe, Canada) and 4D-ISOCT Microscope Imaging System (ISOCT, OptoProbe, Canada).

### 2.5. Determination of the Retinal VEGF-*α*, GFAP, and VCAM-1 mRNA Expression

After blood samples were taken, the left eyes of rats were removed and then the retina tissues were collected at 42 d posttreatment (72 d of total duration of diabetes). Total RNA was extracted from the retinal tissues using Trizol Reagent (Servicebio, G3013, Wuhan, China). RNA concentration was detected by Nanodrop 2000 (Thermo, New York, USA). cDNA was synthesized according to the manufacturer's instructions of RevertAid M-MuLV cDNA synthesis kit (Thermo, #k1622, USA). The fluorescent quantitative polymerase chain reaction (FQ-PCR) was performed using ABI Stepone plus real-time PCR device (New York, USA) according to the protocol of FastStart Universal SYBR-Green Master kit (Roche, Mannheim, Germany). Cycling conditions were as follows: 95°C, 10 min, and 40 cycles of 95°C, 15 s, 60°C, 60 s. The primers of VEGF-*α* were GAGCAGAAAGCCCATGAAGTG (forward) and ACTCCAGGGCTTCATCATTGC (reverse). The primers of GFAP were AGTCGGCGAGTTACCAGGAG (forward) and TTAATGACCTCGCCATCCCG (reverse). The primers of VCAM-1 were TGAACCCAAACAAAGGCAGAGTA (forward) and TTGGGAGTTGGAAAACCATCAC (reverse). The primers of *β*-actin were TGCTATGTTGCCCTAGACTTCG (forward) and GTTGGCATAGAGGTCTTTACGG (reverse). Quantification of VEGF-*α*, GFAP, and VCAM-1 mRNA was normalized to *β*-actin mRNA, and analysis was performed using 2−ΔΔCt method. The final results were expressed as percentage relative to the normal group.

### 2.6. Determination of the Retinal VEGF-*α*, GFAP, and VCAM-1 Protein Expressions

The retinas tissues were separated and homogenized on ice in RIPA buffer (Beyotime, Nantong, Jiangsu Province, China) supplemented with 1:100 of proteinases/phosphatase inhibitor at 42 d posttreatment (72 d of total duration of diabetes) (n=4). Then, the lysates were centrifuged at 12,000 rpm at 4°C for 15 min to obtain the supernatant. A bicinchoninic acid (BCA) protein quantitation kit (Servicebio, Wuhan, China) was applied to determine the concentration of the protein. Equal amount of protein was denatured by boiling in loading sample buffer and then 30 *μ*g protein from each sample was loaded, separated by sodium dodecyl sulfate-polyacrylamide gel electrophoresis using gel (5%, 12%). Next, the proteins were transferred onto PVDF membrane (Millipore, USA) at 120 V for 100 min. The membranes were incubated with 5% non-fat milk solution (Servicebio, Wuhan, China) for 2 h at room temperature and then reacted with VEGF-*α* (1:1000; GB11034, Servicebio, Wuhan, China), GFAP (1:2000; GB11096, Servicebio, Wuhan, China), VCAM-1 (1:1000; 11444-1-AP, Proteintech Group, Wuhan, China), and *β*-actin (1:1000; GB12001, Servicebio, Wuhan, China) at 4°C overnight. The membranes were then incubated with HRP-conjugated secondary antibody (1:30000; GB23303, GB23301; Servicebio, Wuhan, China) at room temperature for 1 h and then enhanced chemiluminescence (Thermo Fisher Scientific, Waltham, MA, US) was used for protein visualization. The intensity of immunoreactivity was quantified by densitometry using Image J software (NIH).

### 2.7. Measurement of Blood Metabolic Indicators

After drug had been administered for 42 days (72 d of total duration of diabetes), fasted (12 h) rats were weighed and anesthetized (1% sodium pentobarbital (Sigma, USA), 3 mL/kg, i.p.). Blood specimens were collected from the vena cava. Serum samples were isolated after centrifugation of whole blood at 3500 r/min at 4°C for 15 min and were stored at −80°C. Enzyme activities of serum antioxidant parameters glutathione peroxidase (GSH-Px), superoxide dismutase (SOD) and catalase (CAT), and levels of serum oxidative stress parameter methane dicarboxylic aldehyde (MDA) and proinflammatory indicator nitric oxide (NO) were assessed using commercially available ultraviolet spectrometry method kits from Nanjing Jiancheng Bioengineering Institute (Nanjing, China), according to the manufacturer's instructions. The levels of serum glucose (GLU), triglycerides (TG), and total cholesterol (TC) were estimated using commercially available kits from Huili Biotech (Changchun, China), according to the manufacturer's instructions. Additionally, advanced glycation end-product formations (AGEs) level was detected by commercially available enzyme-linked immunosorbent assay (ELISA) kits (Cloud-Clone Corp., Wuhan, China), according to the manufacturer's instruction.

### 2.8. Statistical Analysis

All data were expressed as mean ± standard deviation. All statistical analyses were conducted using the SPSS version 19.0 software package (IBM, Chicago, IL, USA). One-way analysis of variance (ANOVA) test was performed, and post hoc multiple comparisons were conducted with LSD.* P* < 0.05 was regarded to be significant.

## 3. Results

### 3.1. Full Field Electroretinography (ffERG)

#### 3.1.1. Full Field Electroretinography (ffERG) before the Treatment

**Figure 1:**
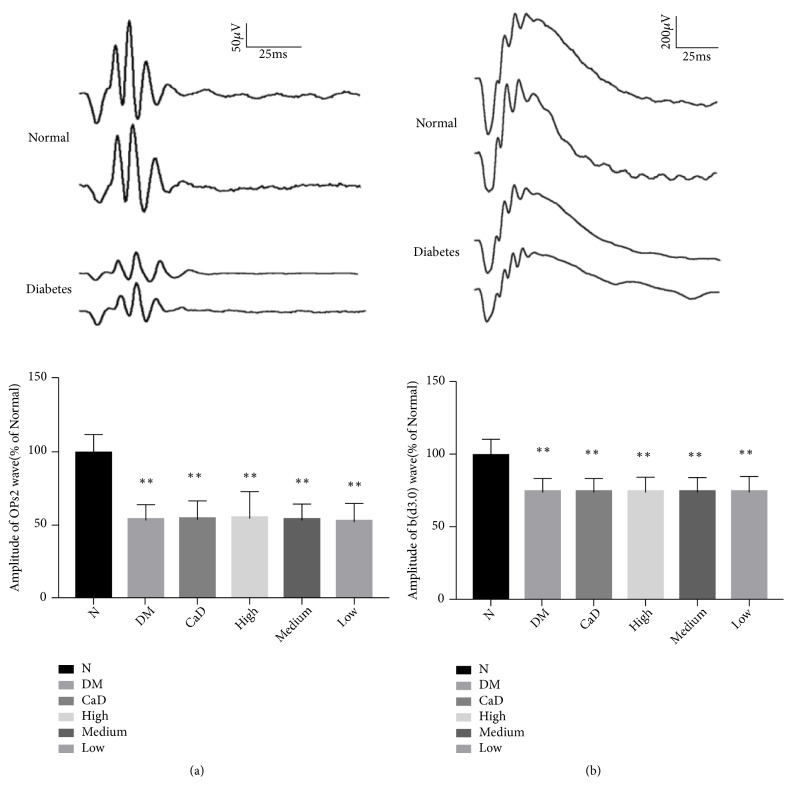
Diabetic rats present decreasing ERG responses after DR rat model established. (a) Dark-adapted OPs2 wave; (b) b-wave in dark-adaptation 3.0 response. Values are presented as mean ± SD, n=10, *∗∗p *< 0.01: untreated diabetic model group, CaD group, FSM high-dose, FSM medium-dose, and FSM low-dose group* vs *normal group.

Full field electroretinography (ffERG) was performed to evaluate the integral retinal function formed by various cell types in the retina. The b wave (dark-adaptation 3.0 response) reflects the electrical activity of cone cell and rod cell. OPs wave indirectly reflects the function of retinal blood vessels. As a typical representative of OPs wave, OPs2 wave can reflect the overall level of OPs wave. At 30 d after diabetic rat model established (before the treatment), as shown in Figure 1, it was found that OPs2 wave decreased to 50-60% of normal eyes (all* p *< 0.01) and b (d3.0) wave decreased to 70-80% of normal eyes (all* p *< 0.01). According to the amplitude of OPs2 wave, diabetic rats were randomly divided into 5 groups as previously described, and the age matching normal SD rats were selected as normal group. The final results were expressed as percentage relative to the normal group.

#### 3.1.2. Full Field Electroretinograms (ffERG) after the Treatment

**Figure 2:**
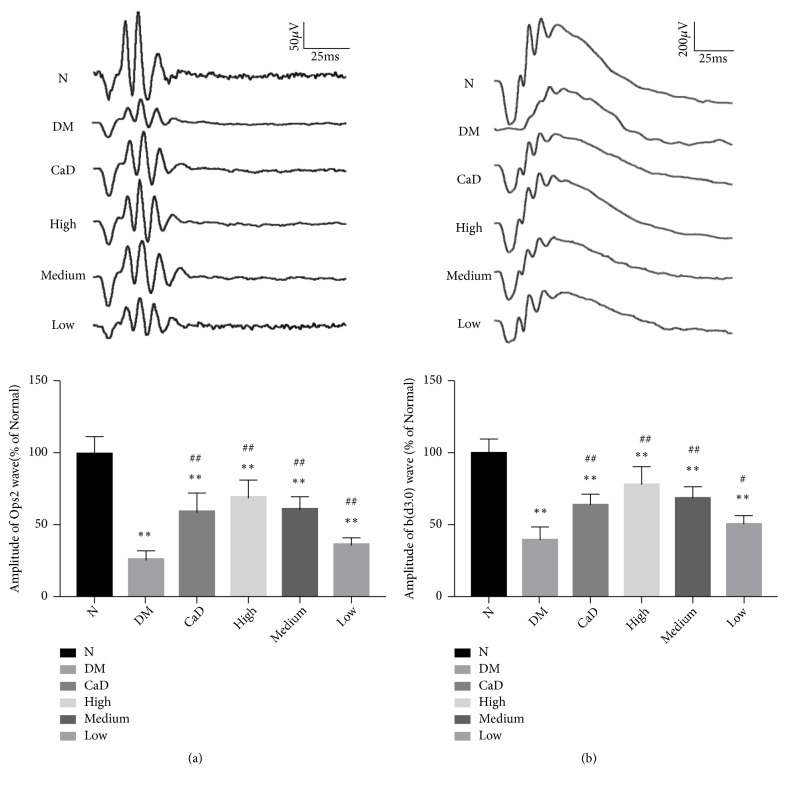
FSM protects diabetic rat ERG after 42-d treatment (72 d of total duration of diabetes). (a) OPs2 wave in different groups; (b) b-wave in dark-adaptation 3.0 response in different groups. Values are presented as mean ± SD, n=8. *∗∗p *< 0.01: untreated diabetic model group, CaD group, FSM high-dose, FSM medium-dose, and FSM low-dose group* vs *normal group; ^#^*p *< 0.05, ^##^*p *< 0.01: CaD group, FSM high-dose, FSM medium-dose, and FSM low-dose group* vs *untreated diabetic model group.

After 42-d treatment (72 d of total duration of diabetes), rats in each group as previously described underwent ERG tests. As shown in Figure 2, compared with normal group, ERG showed that all diabetic group exhibited a significant decreasing amplitude of OPs2 wave (all* p *< 0.01) and b (d3.0) wave (all* p *< 0.01). Notably, in untreated diabetic model group, OPs2 wave decreased to 26% of normal group, and b (d3.0) wave decreased to 40% of normal group. However, CaD group, FSM high-dose, FSM medium-dose, and FSM low-dose group presented an obviously higher amplitude of OPs2 and b (d3.0) wave (OPs2: CaD,* p *< 0.01; FSM high-dose,* p *< 0.01; FSM medium-dose,* p *< 0.01; FSM low-dose,* p *< 0.01; d3.0 response: CaD,* p *< 0.01; FSM high-dose,* p *< 0.01; FSM medium-dose,* p *< 0.01; FSM low-dose,* p *< 0.05) compared with untreated diabetic model group. The final results were expressed as percentage relative to the normal group.

### 3.2. OCT Analysis

#### 3.2.1. OCT Analysis before the Treatment

**Figure 3:**
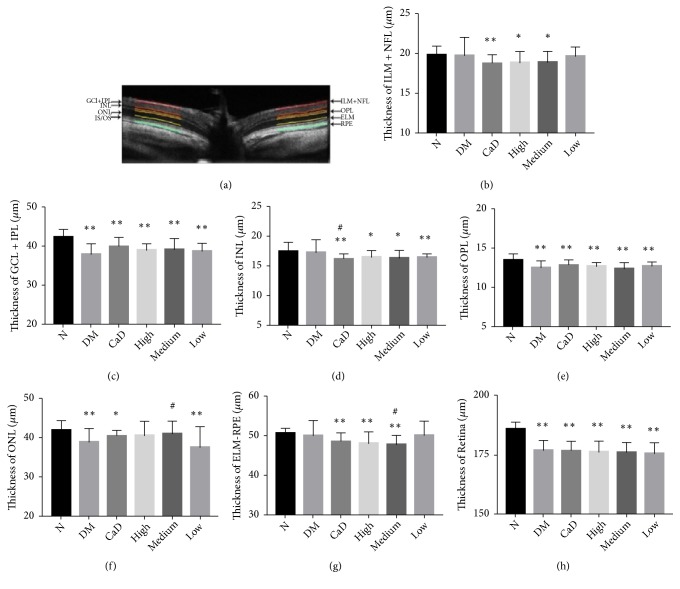
Diabetic rat retinal thickness is decreasing at 30 d after diabetic rat model established (before the treatment). (a) Representative OCT cross-sectional image of retina. (b) The thickness of ILM + NFL; (c) the thickness of GCL + IPL; (d) the thickness of INL; (e) the thickness of OPL; (f) the thickness of ONL; (g) the thickness of EML to RPE; (h) the thickness of entire retina. ILM+NFL: internal limiting membrane and nerve fiber layer; GCL + IPL: ganglion cell layer and inner plexiform layer; INL: inner nuclear layer; OPL: outer plexiform layer; ONL: outer nuclear layer; EML to RPE: external limiting membrane to retinal pigment epithelium. Values are presented as mean ± SD, n=10, *∗p *< 0.05, *∗∗p *< 0.01: untreated diabetic model group, CaD group, FSM high-dose, FSM medium-dose, and FSM low-dose group* vs* normal group;^ #^*p *< 0.05: CaD group, FSM high-dose, FSM medium-dose, and FSM low-dose group* vs* untreated diabetic model group.

OCT was applied to evaluate retinal morphometric structure. As shown in Figure 3, the total retinal thickness decreased significantly at 30 d after diabetic rat model established. Particularly, ganglion cell layer (GCL) and inner plexiform layer (IPL), outer plexiform layer (OPL), and entire retina thickness became thinner (all* p *< 0.01). Thus, diabetic rats exhibited a thinner retinal thickness, which indicated to some extent that the retinal structure alterations occurred.

#### 3.2.2. OCT Analysis after the Treatment

**Figure 4:**
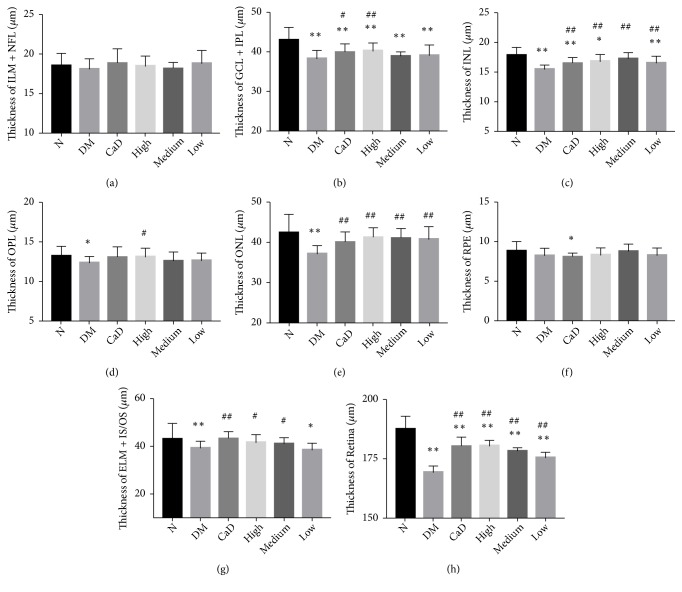
FSM protects diabetic rat retinal morphometric structure after 42-d treatment (72 d of total duration of diabetes). (a) The thickness of ILM & NFL; (b) the thickness of GCL + IPL; (c) the thickness of INL; (d) the thickness of OPL; (e) the thickness of ONL; (f) the thickness of RPE; (g) the thickness of EML + IS/OS; (h) the thickness of entire retina. ILM+NFL: internal limiting membrane and nerve fiber layer; GCL + IPL: ganglion cell layer and inner plexiform layer; INL: inner nuclear layer; OPL: outer plexiform layer; ONL: outer nuclear layer; RPE: retinal pigment epithelium; IS/OS + ELM: photoreceptor inner segment/outer segment and external limiting membrane. Values are presented as mean ± SD, n=8. *∗p *< 0.05, *∗∗p *< 0.01: untreated diabetic model group, CaD group, FSM high-dose, FSM medium-dose, and FSM low-dose group* vs *normal group; ^#^*p *< 0.05, ^##^*p *< 0.01: CaD group, FSM high-dose, FSM medium-dose, and FSM low-dose group* vs *untreated diabetic model group.

OCT was examined to observe retinal structural alterations after 42-d treatment (72 d of total duration of diabetes). As shown in Figure 4, the retina thickness of diabetic rats, especially, ganglion cell layer and inner plexiform layer (GCL and IPL), inner nuclear layer (INL), outer nuclear layer (ONL), and entire retinal thickness were declining compared with normal group (all* p *< 0.05). Additionally, CaD group, FSM high-dose, FSM medium-dose, and FSM low-dose group showed a lower decrease in INL, ONL, and total retinal thickness (all* p *< 0.01) compared with untreated diabetic rats. Moreover, CaD group and FSM high-dose group had thicker GCL and IPL thickness than untreated diabetic rats (all* p *< 0.01). Besides, CaD group, FSM high-dose, and FSM medium-dose group showed thicker external limiting membrane (ELM) and photoreceptor inner/outer segment (IS/OS) thickness than untreated diabetic rats (all* p *< 0.05). FSM and CaD both showed protective effect on retinal structure integrity. As for the entire thickness of internal limiting membrane (ILM) and nerve fiber layer (NFL), there existed no significant difference among different groups (all* p *> 0.05).

### 3.3. VEGF-*α*, GFAP, and VCAM-1 mRNA Expressions

**Figure 5:**
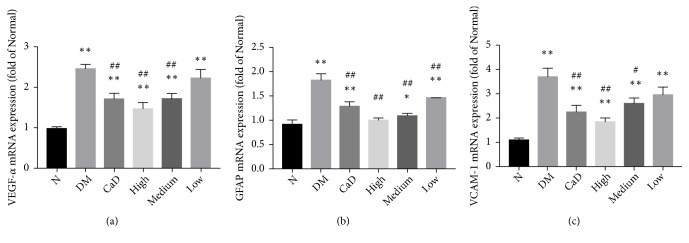
FSM decreases diabetic rat retinal VEGF-*α*, GFAP, and VCAM-1 mRNA levels after 42-d treatment (72 d of total duration of diabetes). The mRNA levels were assessed by FQ-PCR. (a) Values of VEGF-*α*. (b) Values of GFAP. (c) Values of VCAM-1. Data are presented as percentage of control and values are presented as mean ± SD, n=3-4. *∗p *< 0.05, *∗∗p *< 0.01: untreated diabetic model group, CaD group, FSM high-dose, FSM medium-dose, and FSM low-dose group* vs *normal group; ^#^*p *< 0.05, ^##^*p *< 0.01: CaD group, FSM high-dose, FSM medium-dose, and FSM low-dose group* vs *untreated diabetic model group.

In order to evaluate the potential anti-inflammatory properties and effect on glial cell reactivity, VEGF-*α*, GFAP, and VCAM-1 mRNA levels were analyzed by FQ-PCR after 42-d treatment (72 d of total duration of diabetes). As is shown in Figure 5, the expressions of VEGF-*α*, GFAP, and VCAM-1 in all diabetic groups, excepting the expression of GFAP in FSM high-dose group, were incredibly more than those in normal group (all* p *< 0.05). Nevertheless, the expression of GFAP in CaD group, FSM high-dose, FSM medium-dose, and FSM low-dose group was decreasing compared with that in untreated diabetic model group (all* p *< 0.05). Moreover, the expressions of VEGF-*α* and VCAM-1 in CaD group, FSM high-dose, and FSM medium-dose group were less than those in untreated diabetic model group (all* p *< 0.05).

### 3.4. VEGF-*α*, GFAP, and VCAM-1 Protein Expressions

**Figure 6:**
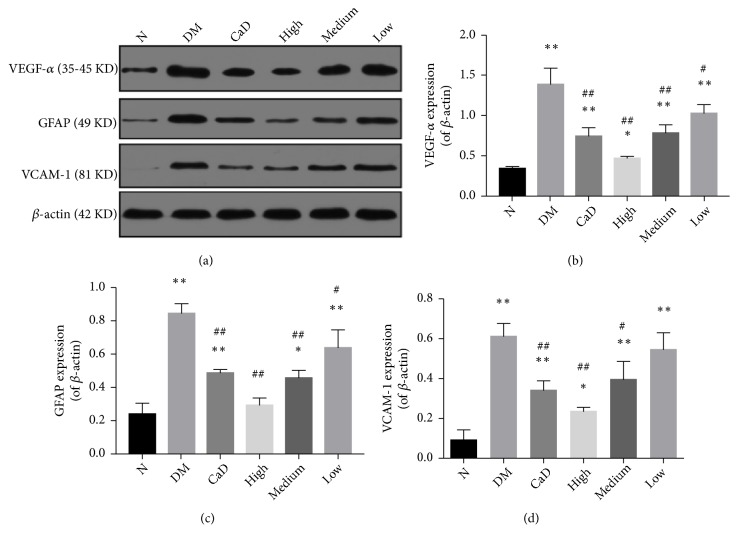
FSM decreases diabetic rat retinal VEGF-*α*, GFAP, and VCAM-1 protein levels after 42-d treatment (72 d of total duration of diabetes). (a) VEGF-*α*, GFAP, and VCAM-1 representative western blots, with the respective loading control (*β*-actin); (b) relative density of immunoblot of VEGF-*α*; (c) relative density of immunoblot of GFAP; (d) relative density of immunoblot of Vcam-1. Values are presented as mean ± SD, n=3-4. *∗p *< 0.05, *∗∗p *< 0.01: untreated diabetic model group, CaD group, FSM high-dose, FSM medium-dose, and FSM low-dose group* vs *normal group; ^#^*p *< 0.05, ^##^*p *< 0.01: CaD group, FSM high-dose, FSM medium-dose, and FSM low-dose group* vs *untreated diabetic model group.

After 42-d treatment (72 d of total duration of diabetes), proinflammatory molecular VEGF-*α*, GFAP, and Vcam-1 protein expressions were detected by western blot method. As is shown in Figure 6, the expressions of VEGF-*α*, GFAP, and VCAM-1 in all diabetic groups, excepting the expression of GFAP in FSM high-dose group, were notably more than those in normal group, in agreement with the results obtained by FQ-PCR. However, the expressions of VEGF-*α* and GFAP in CaD group, FSM high-dose, FSM medium-dose, and FSM low-dose group were less than those in untreated diabetic model group (all* p *< 0.05). Additionally, the expression of VCAM-1 in CaD group and FSM high-dose and FSM medium-dose groups was less than that in untreated diabetic model group (all* p *< 0.05), in consistence with the results obtained by FQ-PCR.

### 3.5. Blood Metabolic Indicators 

#### 3.5.1. The Level of GLU, TC, and TG

**Figure 7:**
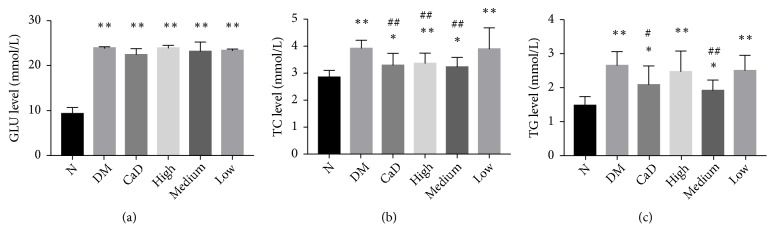
Effects of FSM on serum level of GLU, TC, and TG after 42-d treatment (72 d of total duration of diabetes). (a) The serum level of GLU level; (b) the serum level of TC; (c) the serum level of TG. Values are presented as mean ± SD, n=8. *∗p *< 0.05, *∗∗p *< 0.01: untreated diabetic model group, CaD group, FSM high-dose, FSM medium-dose, and FSM low-dose group* vs *normal group; ^#^*p *< 0.05, ^##^*p *< 0.01: CaD group, FSM high-dose, FSM medium-dose, and FSM low-dose group* vs *untreated diabetic model group.

As compared with normal rats, serum levels of GLU, TC, and TG in diabetic rats were markedly increased (all* p *< 0.05) (Figure 7). There is no significant difference in GLU among diabetic groups (all* p* > 0.05). After 42-d treatment (72 d of total duration of diabetes), CaD group, FSM high-dose group, and FSM medium-dose group notably decreased TC level (all* p *< 0.01 versus the untreated diabetic model group). Additionally, CaD group and FSM medium-dose group significantly decreased TG level (all* p *< 0. 05 versus the untreated diabetic model group).

#### 3.5.2. The Level of Detrimental Accumulation of AGEs, MDA, and NO

**Figure 8:**
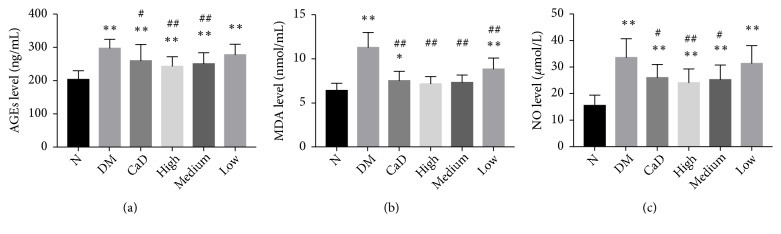
FSM downregulates the detrimental accumulation of AGEs, MDA, and NO after 42-d treatment (72 d of total duration of diabetes). (a) The serum level of AGEs; (b) the serum level of MDA; (c) the serum level of NO. Values are presented as mean ± SD, n=8. *∗p *< 0.05, *∗∗p *< 0.01: untreated diabetic model group, CaD group, FSM high-dose, FSM medium-dose, and FSM low-dose group* vs *normal group; ^#^*p *< 0.05, ^##^*p *< 0.01: CaD group, FSM high-dose, FSM medium-dose, and FSM low-dose group* vs *untreated diabetic model group.

Serum AGEs, MDA, and NO levels were measured to assess the degree of oxidative stress and inflammatory condition/nitrosative stress in rats after 42-d treatment (72 d of total duration of diabetes). As compared with normal rats, serum levels of AGEs, MDA, and NO in diabetic rats, excepting the levels of MDA in FSM high-dose and medium-dose group, were markedly increased (all *p* < 0.05) (Figure 8). As compared with untreated diabetic model group, CaD group, FSM high-dose group, and FSM medium-dose group notably decreased AGEs and NO levels (all *p* < 0.05). Additionally, CaD group, FSM high-dose, FSM medium-dose, and FSM low-dose group notably decreased MDA level (all *p* < 0.01 versus the untreated diabetic model group).

#### 3.5.3. The Activity of Antioxidant Enzymes GSH-Px, SOD, and CAT

**Figure 9:**
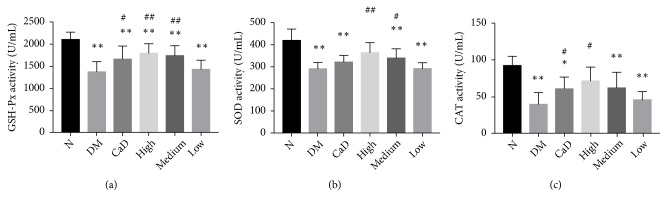
FSM upregulates the activity of serum antioxidant enzyme GSH-Px, SOD, and CAT after 42-d treatment (72 d of total duration of diabetes). (a) The serum activity of GSH-Px; (b) the serum activity of SOD; (c) the serum activity of CAT. Values are presented as mean ± SD, n=8. *∗p *< 0.05, *∗∗p *< 0.01: untreated diabetic model group, CaD group, FSM high-dose, FSM medium-dose, and FSM low-dose group* vs *normal group; ^#^*p *< 0.05, ^##^*p *< 0.01: CaD group, FSM high-dose, FSM medium-dose, and FSM low-dose group* vs *untreated diabetic model group.

Considering the antioxidant stress property of FSM, the effects of this compound were evaluated on serum GSH-Px, SOD, and CAT activities after 42-d treatment (72 d of total duration of diabetes). As compared with normal rats, serum levels of GSH-Px, SOD, and CAT in diabetic rats, excepting the levels of SOD and CAT in FSM high-dose group, were markedly decreased (all *p* < 0.05) (Figure 9). However, CaD group, FSM high-dose, and FSM medium-dose group notably increased GSH-Px level (all *p* < 0.05 versus the untreated diabetic model group). FSM high-dose and FSM medium-dose group significantly increased SOD level (all *p* < 0.05 versus the untreated diabetic model group). Additionally, CaD group and FSM high-dose group significantly decreased CAT level (all *p* < 0.05 versus the untreated diabetic model group).

## 4. Discussion

Diabetic retinopathy (DR) is the leading cause of visual impairment and preventable blindness [1], which represents a significant socioeconomic burden for healthcare systems worldwide [7]. In the study, we evaluated the effect of compound traditional Chinese medicine FSM on DR treatment. A better retinal function and structure were found in FSM treatment groups because of an enhanced ability of antioxidation and anti-inflammation.

In the present study, diabetes was established by 6-week high-fat and high-sugar diet combined with 35 mg/kg streptozotocin (STZ) injection. We found that, at 30 days post diabetic rat model established, retinal function in the diabetic rats was decreased, both b (d3.0) wave and OPs2 wave compared with normal rats. Furthermore, diabetic rats were randomly grouped by OPs2 indicator. It is reported that oscillatory potentials (OPs) are considered the most relevant ERG test for DR diagnosis and progression [43]. Clinical research found that a frequent reduction or absence of OPs was shown in diabetic patients even in a preclinical stage of retinopathy, and the lower the amplitude of OPs2 is, the severer the DR is [44]. Otherwise, the reduction of retina thickness showed in OCT affirmed the retina morphological alterations. After 42-day treatment, retinal function in the FSM treated groups was restored in both b wave and OPs which reflected functions of on & off bipolar cells and the vascular [45], compared with those of the untreated diabetic model group. Thus, FSM could rescue the retinal function and protect the function of retinal blood vessels. Additionally, FSM could significantly attenuate retinal structures damage induced by diabetes. Specifically, the thicknesses of INL, ONL, and entire retina in FSM groups were less decreasing, which revealed that the protective effect of FSM on the retinal structure contributed to resisting the retinal cells electrical activity decline.

Our results suggested a vascular endothelial dysfunction in diabetic rat retina, as estimated by assays of VCAM-1 protein and VCAM-1 mRNA expression. It was found that the upregulation of VCAM-1, a cell adhesion molecule, associated to increased inflammation-related genes transcription such as tumor necrosis factor-alpha (TNF-*α*), interleukin-1 (IL-1), and nuclear factor-kappa B (NF-*κ*B) [46]. Thus, we speculated that the enhanced endothelial activation reflected the inflammatory nature in this hyperglycemic state [47]. Interestingly, hyperglycemia also caused the stress condition of astrocytes and Müller cells, which was suggested by the increased expression of retinal GFAP in DR rats and these findings were in agreement with previous studies [38, 48]. The induction of GFAP expression usually occurred after retinal damage and indicated that the retina was undergoing degeneration [49]. It was worth mentioning that the upregulation of GFAP also initiated proinflammatory cytokines release [50]. Our findings suggested that FSM and CaD could relieve the inflammation by inhibiting the increase of VCAM-1 and GFAP expression. Furthermore, FSM and CaD reduced the upregulated expression of retinal VEGF-*α*, a major angiogenic factor, in DR rats. VEGF-*α* was considered to associate with oxidative stress and inflammatory response and induce retinal neovascularization, vascular leakage, and macular edema [51–53]. Additionally, the effect of CaD on VEGF-*α* was confirmed by other researches [54, 55], but FSM probably revealed better effects, at least according to the PCR and western blot results in our research. Our results presented that the compound traditional Chinese medicine FSM could downregulate the expression of VEGF-*α* to depress angiogenesis of DR, which may provide a new option or supplement for patients.

It was noteworthy that diabetic rats showed not only upregulation of inflammatory mediators and endothelial activation in the retina, but also a systemic inflammatory response and oxidative stress in this model. It was reported that high serum NO production would increase systemic oxidative damage and nitrosative stress, which was related to increased severity of DR [18, 56, 57]. Moreover, upregulation of ROS could lead to enhanced activation of NF-kB, which, in turn, increased the release of proinflammatory cytokines and NO. Therefore, oxidative stress might indirectly contribute to DR through stimulating inflammation or directly contribute to the DR via oxidative damage to cells. Additionally, MDA, one of the breakdown products of peroxidized polyunsaturated fatty acids, was considered to be sensitive and reliable for the assessment of oxidative stress [58, 59]. AGEs accumulated under hyperglycemic conditions were reported to increase proinflammatory mediators, stimulate ROS production, cause retinal microvascular endothelial cells dysfunction, and enhance NO and VEGF secretion. They all occurred against a background of the various metabolic derangements that were inherent to diabetes [60]. Our results revealed that, compared with untreated diabetes, serum NO, MDA, and AGEs concentrations were downregulated and antioxidative SOD, GSH-Px, and CAT activities were upregulated by FSM, which indicated that FSM could protect the retinal structure and function via regulating nitrogen species homeostasis, reducing oxidative stress, and ameliorating inflammatory response.

Moreover, dyslipidemia was found in DR rats. Long-term effects of dyslipidemia might be of importance for progression of DR and it was associated with retinal hard exudate and visual loss [61]. Reducing the levels of serum triglycerides showed an effect on the progression of DR, as reported in several observation studies [1]. In consistence with those reports, our data supported the fact that TC and TG levels were increased in DR rats. Furthermore, FSM depressed serum TC and TG level, and this might be related to the hypolipidemic activity of puerarin, the main compound constituting FSM [8], which could rapidly be absorbed in the small intestines [62] and increase the fecal excretion of TG, promote the oxidation of fatty acids, and suppress the synthesis of fats [63].

In this study, we speculated that the active ingredients puerarin, ginsenosides Rb1, ligustilide, aurantio obtusin, and chrysophanol of FSM could protect retinal structure and promote retinal function via sustaining a better retinal microenvironment due to their anti-inflammatory, antioxidative, and anticoagulating abilities, which was proved by numerous researches [9–28]. Interestingly, puerarin was also reported to inhibit IL-1*β*-induced leukostasis by regulating the expression of VCAM-1 [14], and this at least partially supported our findings.

Our findings not only revealed the effects of FSM on retinal damage but also determined the pharmacological effect of CaD that was confirmed by other studies, and our results were consistent with those previous reports about CaD. In this regard, FSM showed satisfying benefits on protecting retinal function and structure and blood homeostasis, which resembled or rivaled CaD. However, lack of research on further mechanism of the major compounds of FSM may be the limitation in our study. In the next step, we would like to thoroughly evaluate the accurate mechanisms of the components of FSM on DR.

## 5. Conclusion

In summary, FSM exerted a multifaceted action on protecting the diabetic rat retinal function and morphological structure integrity. The potential mechanism of FSM protective role would be associated with antioxidant effects directly by scavenging free radicals or indirectly by increasing the antioxidant enzymes, regulating nitrogen species homeostasis, and ameliorating inflammatory response. Therefore, FSM could be contemplated as an alternative candidate for treatment of DR.

## Data Availability

The original data used to support the findings of this study are included within the article.
